# Supplementary material for: Electronic based reported anthropometry—A useful tool for interim monitoring of obesity prevalence in developing states
Source: PLoS One. 2020 Dec 7;15(12):e0243202. doi: 10.1371/journal.pone.0243202 (PMC7721176; doi:10.1371/journal.pone.0243202)
Supplement: S1 Questionnaire — (DOCX) [file pone.0243202.s001.docx]

**Barbados Children’s Health and Nutrition Study**

**The St. Michael School Canteen Pilot**

**To be answered by parents:**

I agree to allow my child to take part in the Barbados Children’s Health and Nutrition Study:

- 1. I agree
  2. No, I do not agree

**Please note that these questions are confidential. Neither peers or others will be able to see your answers.**

1. Child ID Number:
2. SEX: {M/F}
3. Date of birth: {dd/mm/yyyy}
4. Date: {dd/mm/yyyy}
5. Email address:
6. What is your current weight? {lb/kg}
7. What is your current height? {ft. in./cm}
8. What Form are you in?
9. Who do you live with?
10. How many adults over 18 years of age are living in your household?
11. How many children less than 18 years of age live in your household? Please count yourself if you are less than 18 year.
12. How many rooms are there in your house? (do not count your kitchen or bathroom(s))
13. Does anyone in the household own a vehicle? (Car, van, etc.)
14. Did you buy food form the Canteen LAST TERM ?
15. Why did you not buy food from the Canteen Last Term?
16. What meal(s) did you eat from the canteen Last Term?
17. Do you buy food form the Canteen NOW ?
18. Why do you not buy food from the Canteen NOW ?
19. What meals do you eat from the canteen? **(Please choose all that apply)**
    1. {Breakfast, Morning snack, Lunch, Evening snack}

Foods offered by the canteen:

1. **Fresh fruit**:
   1. How many times a week do you buy it? {Open end}
   2. I buy **Fresh fruit** because:
      1. It is cheap
      2. I like it
   3. I DO NOT buy **Fresh fruit** because:
      1. It is expensive
      2. I do not like it
      3. It is unhealthy
      4. Allergies/intolerance
      5. Religious reasons
2. **Ham and cheese croissants**:
   1. How many times a week do you buy it? {Open end}
   2. I buy **Ham and cheese croissants** because:
      1. It is cheap
      2. I like it
   3. I DO NOT buy **Ham and cheese croissants** because:
      1. It is expensive
      2. I do not like it
      3. It is unhealthy
      4. Allergies/intolerance
      5. Religious reason
3. **Muffins**:
4. How many times a week do you buy it? {Open end}
5. I buy **Muffins** because:
   - 1. It is cheap
     2. I like it
6. I DO NOT buy **Muffins** because:
   - 1. It is expensive
     2. I do not like it
     3. It is unhealthy
     4. Allergies/intolerance
     5. Religious reasons
7. **Pancakes**:
8. How many times a week do you buy it? {Open end}
9. I buy **Pancakes** because:
   - 1. It is cheap
     2. I like it
10. I DO NOT buy **Pancakes** because:
    - 1. It is expensive
      2. I do not like it
      3. It is unhealthy
      4. Allergies/intolerance
      5. Religious reasons
11. **Omelette and toast**:
12. How many times a week do you buy it? {Open end}
13. I buy **Omelette and toast** because:
    - 1. It is cheap
      2. I like it
14. I DO NOT buy **Omelette and toast** because:
    - 1. It is expensive
      2. I do not like it
      3. It is unhealthy
      4. Allergies/intolerance
      5. Religious reasons
15. **Pizza**:
16. How many times a week do you buy it? {Open end}
17. I buy **Pizza** because:
    - 1. It is cheap
      2. I like it
18. I DO NOT buy **Pizza** because:
    - 1. It is expensive
      2. I do not like it
      3. It is unhealthy
      4. Allergies/intolerance
      5. Religious reasons
19. **Hot dogs**:
20. How many times a week do you buy it? {Open end}
21. I buy **Hot dogs** because:
    - 1. It is cheap
      2. I like it
22. I DO NOT buy **Hot dogs** because:
    - 1. It is expensive
      2. I do not like it
      3. It is unhealthy
      4. Allergies/intolerance
      5. Religious reasons
23. **Hamburgers**:
24. How many times a week do you buy it? {Open end}
25. I buy **Hamburgers** because:
    - 1. It is cheap
      2. I like it
26. I DO NOT buy **Hamburgers** because:
    - 1. It is expensive
      2. I do not like it
      3. It is unhealthy
      4. Allergies/intolerance
      5. Religious reasons
27. **Rotis**:
28. How many times a week do you buy it? {Open end}
29. I buy **Rotis** because:
    - 1. It is cheap
      2. I like it
30. I DO NOT buy **Rotis** because:
    - 1. It is expensive
      2. I do not like it
      3. It is unhealthy
      4. Allergies/intolerance
      5. Religious reasons
31. **Chicken/fish wraps**:
32. How many times a week do you buy it? {Open end}
33. I buy **Chicken/fish wraps** because:
    - 1. It is cheap
      2. I like it
34. I DO NOT buy **Chicken/fish wraps** because:
    - 1. It is expensive
      2. I do not like it
      3. It is unhealthy
      4. Allergies/intolerance
      5. Religious reasons
35. **Garden salad**:
36. How many times a week do you buy it? {Open end}
37. I buy **Garden salad** because:
    - 1. It is cheap
      2. I like it
38. I DO NOT buy **Garden salad** because:
    - 1. It is expensive
      2. I do not like it
      3. It is unhealthy
      4. Allergies/intolerance
      5. Religious reasons
39. **Big frank snacks**:
40. How many times a week do you buy it? {Open end}
41. I buy **Big frank snacks** because:
    - 1. It is cheap
      2. I like it
42. I DO NOT buy **Big frank snacks** because:
    - 1. It is expensive
      2. I do not like it
      3. It is unhealthy
      4. Allergies/intolerance
      5. Religious reasons
43. **Macaroni pie**:
44. How many times a week do you buy it? {Open end}
45. I buy **Macaroni pie** because:
    - 1. It is cheap
      2. I like it
46. I DO NOT buy **Macaroni pie** because:
    - 1. It is expensive
      2. I do not like it
      3. It is unhealthy
      4. Allergies/intolerance
      5. Religious reasons
47. **Chips**:
48. How many times a week do you buy it? {Open end}
49. I buy **Chips** because:
    - 1. It is cheap
      2. I like it
50. I DO NOT buy **Chips** because:
    - 1. It is expensive
      2. I do not like it
      3. It is unhealthy
      4. Allergies/intolerance
      5. Religious reasons
51. **Soup**:
52. How many times a week do you buy it? {Open end}
53. I buy **Soup** because:
    - 1. It is cheap
      2. I like it
54. I DO NOT buy **Soup** because:
    - 1. It is expensive
      2. I do not like it
      3. It is unhealthy
      4. Allergies/intolerance
      5. Religious reasons
55. **Chicken alfredo**:
56. How many times a week do you buy it? {Open end}
57. I buy **Chicken alfredo** because:
    - 1. It is cheap
      2. I like it
58. I DO NOT buy **Chicken alfredo** because:
    - 1. It is expensive
      2. I do not like it
      3. It is unhealthy
      4. Allergies/intolerance
      5. Religious reasons
59. **Chicken**:
60. How many times a week do you buy it? {Open end}
61. I buy **Chicken** because:
    - 1. It is cheap
      2. I like it
62. I DO NOT buy **Chicken** because:
    - 1. It is expensive
      2. I do not like it
      3. It is unhealthy
      4. Allergies/intolerance
      5. Religious reasons
63. **Bottled water**:
64. How many times a week do you buy it? {Open end}
65. I buy **Bottled water** because:
    - 1. It is cheap
      2. I like it
66. I DO NOT buy **Bottled water** because:
    - 1. It is expensive
      2. I do not like it
      3. It is unhealthy
      4. Allergies/intolerance
      5. Religious reasons
67. **Juices**:
68. How many times a week do you buy it? {Open end}
69. I buy **Juices** because:
    - 1. It is cheap
      2. I like it
70. I DO NOT buy **Juices** because:
    - 1. It is expensive
      2. I do not like it
      3. It is unhealthy
      4. Allergies/intolerance
      5. Religious reasons
71. **Soft drinks**:
72. How many times a week do you buy it? {Open end}
73. I buy **Soft drinks** because:
    - 1. It is cheap
      2. I like it
74. I DO NOT buy **Soft drinks** because:
    - 1. It is expensive
      2. I do not like it
      3. It is unhealthy
      4. Allergies/intolerance
      5. Religious reasons
75. **Cran water**:
76. How many times a week do you buy it? {Open end}
77. I buy **Cran water** because:
    - 1. It is cheap
      2. I like it
78. I DO NOT buy **Cran water** because:
    - 1. It is expensive
      2. I do not like it
      3. It is unhealthy
      4. Allergies/intolerance
      5. Religious reasons
79. **Mauby**:
80. How many times a week do you buy it? {Open end}
81. I buy **Mauby** because:
    - 1. It is cheap
      2. I like it
82. I DO NOT buy **Mauby** because:
    - 1. It is expensive
      2. I do not like it
      3. It is unhealthy
      4. Allergies/intolerance
      5. Religious reasons
83. **Lemonade**:
84. How many times a week do you buy it? {Open end}
85. I buy **Lemonade** because:
    - 1. It is cheap
      2. I like it
86. I DO NOT buy **Lemonade** because:
    - 1. It is expensive
      2. I do not like it
      3. It is unhealthy
      4. Allergies/intolerance
      5. Religious reasons
87. **Powerade**:
88. How many times a week do you buy it? {Open end}
89. I buy **Powerade** because:
    - 1. It is cheap
      2. I like it
90. I DO NOT buy **Powerade** because:
    - 1. It is expensive
      2. I do not like it
      3. It is unhealthy
      4. Allergies/intolerance
      5. Religious reasons
91. **Cookies**:
92. How many times a week do you buy it? {Open end}
93. I buy **Cookies** because:
    - 1. It is cheap
      2. I like it
94. I DO NOT buy **Cookies** because:
    - 1. It is expensive
      2. I do not like it
      3. It is unhealthy
      4. Allergies/intolerance
      5. Religious reasons
95. **Brownies**:
96. How many times a week do you buy it? {Open end}
97. I buy **Brownies** because:
    - 1. It is cheap
      2. I like it
98. I DO NOT buy **Brownies** because:
    - 1. It is expensive
      2. I do not like it
      3. It is unhealthy
      4. Allergies/intolerance
      5. Religious reasons
99. **Fruit cups**:
100. How many times a week do you buy it? {Open end}
101. I buy **Fruit cups** because:
     - 1. It is cheap
       2. I like it
102. I DO NOT buy **Fruit cups** because:
     - 1. It is expensive
       2. I do not like it
       3. It is unhealthy
       4. Allergies/intolerance
       5. Religious reasons
103. **Fruit snacks**:
104. How many times a week do you buy it? {Open end}
105. I buy **Fruit snacks** because:
     - 1. It is cheap
       2. I like it
106. I DO NOT buy **Fruit snacks** because:
     - 1. It is expensive
       2. I do not like it
       3. It is unhealthy
       4. Allergies/intolerance
       5. Religious reasons
107. **Fruit salad**:
108. How many times a week do you buy it? {Open end}
109. I buy **Fruit salad** because:
     - 1. It is cheap
       2. I like it
110. I DO NOT buy **Fruit salad** because:
     - 1. It is expensive
       2. I do not like it
       3. It is unhealthy
       4. Allergies/intolerance
       5. Religious reasons
111. **Jampuffs**:
112. How many times a week do you buy it? {Open end}
113. I buy **Jampuffs** because:
     - 1. It is cheap
       2. I like it
114. I DO NOT buy **Jampuffs** because:
     - 1. It is expensive
       2. I do not like it
       3. It is unhealthy
       4. Allergies/intolerance
       5. Religious reasons
115. **Turnovers**:
116. How many times a week do you buy it? {Open end}
117. I buy **Turnovers** because:
     - 1. It is cheap
       2. I like it
118. I DO NOT buy **Turnovers** because:
     - 1. It is expensive
       2. I do not like it
       3. It is unhealthy
       4. Allergies/intolerance
       5. Religious reasons
119. **Spaghetti Bolognese**:
120. How many times a week do you buy it? {Open end}
121. I buy **Spaghetti Bolognese** because:
     - 1. It is cheap
       2. I like it
122. I DO NOT buy **Spaghetti Bolognese** because:
     - 1. It is expensive
       2. I do not like it
       3. It is unhealthy
       4. Allergies/intolerance
       5. Religious reasons
123. **Lasagna**:
124. How many times a week do you buy it? {Open end}
125. I buy **Lasagna** because:
     - 1. It is cheap
       2. I like it
126. I DO NOT buy **Lasagna** because:
     - 1. It is expensive
       2. I do not like it
       3. It is unhealthy
       4. Allergies/intolerance
       5. Religious reasons
127. How much do you enjoy the food that is served at the canteen NOW?
     1. 1 star
     2. 2 stars
     3. 3 stars
     4. 4 stars
     5. 5 stars
128. Is there anything you would like the canteen to do to improve its food or service. Write your comment in the box below.
129. Did you prefer the foods offered by the previous canteen operator?

{Y/N/No preference}

1. How much did you enjoy the food that was served at the canteen Last semester?
   1. 1 star
   2. 2 stars
   3. 3 stars
   4. 4 stars
   5. 5 stars
