# Supplementary material for: Electronic based reported anthropometry—A useful tool for interim monitoring of obesity prevalence in developing states
Source: PLoS One. 2020 Dec 7;15(12):e0243202. doi: 10.1371/journal.pone.0243202 (PMC7721176; doi:10.1371/journal.pone.0243202)
Supplement: S1 File — (DOC) [file pone.0243202.s002.doc]

**The Barbados Children’s Health and Nutrition Study**

**The St. Michael School Canteen Pilot**

**CHILD PARTICIPANT ASSENT**

I understand that my height and weight will be measured by a trained person associated with this study. Also, my personal information will not be given to unauthorized persons.

***Check whether or not you want to participate in this research study:***

[ ] Yes, I want to participate [ ] No, I don’t want to participate

_______________________________ _____________________ __________________

Child’s Name Person obtaining assent Date

**___ ___ ___**

**…………………………………………………………………………………………………………………**

**The Barbados Children’s Health and Nutrition Study (BCHNS)**

**CHILD PARTICIPANT ASSENT**

I understand that my height and weight will be measured by a trained person associated with this study. Also, my personal information will not be given to unauthorized persons.

***Check whether or not you want to participate in this research study:***

[ ] Yes, I want to participate [ ] No, I don’t want to participate

_______________________________ _____________________ __________________

Child’s Name Person obtaining assent Date

**___ ___ ___**
