# Supplementary material for: Electronic based reported anthropometry—A useful tool for interim monitoring of obesity prevalence in developing states
Source: PLoS One. 2020 Dec 7;15(12):e0243202. doi: 10.1371/journal.pone.0243202 (PMC7721176; doi:10.1371/journal.pone.0243202)
Supplement: S2 File — (DOCX) [file pone.0243202.s003.docx]

| **St. Michael's Canteen Study Electronic Survey Proforma** | | |  |
| --- | --- | --- | --- |
| *Item* | Number | Percentage |  |
|  |  |  |  |
| Total number of students | 790 |  |  |
| Number emailed | 790 |  |  |
| Number who responded | 71 | 9% |  |
|  |  |  |  |
| Number of complete questionnaires | 35 | 49% |  |
| Number of incomplete questionnaires | 36 | 51% |  |
| Number of questionnaires terminated by question 17 (43) | 28 | 78% |  |
| Number with sex correct | 71 | 100% |  |
| Number with incorrect sex | 0 | 0% |  |
| Number with date of birth correct | 70 | 99% |  |
| Number with date of birth incorrect | 1 | 1% |  |
| Number with form correct | 71 | 100% |  |
| Number with form incorrect | 0 | 0% |  |
| Number who reported weight in kilograms | 9 | 13% |  |
| Number who reported weight in pounds | 58 | 82% |  |
| Number who gave no response to weight question | 4 | 6% |  |
| Number who used the weight units incorrectly | 1 | 1% |  |
| Number who reported height in centimeters | 16 | 23% |  |
| Number who reported height in feet and inches | 46 | 65% |  |
| Number who gave no response to height question | 7 | 10% |  |
| Number who used the height units incorrectly | 3 | 4% |  |
| Total number of responses with menu options | 4189 | 100% |  |
| Number of responses using drop-down menu | 3858 | 92% |  |
| Number of items with appropriate skip patterns | 4189 | 100% |  |
| Number of items with inappropriate skip patterns | 0 | 0% |  |
| Number of open-ended responses | 331 | 8% |  |
| Number of open ended responses with similar answers provided in the dropdown menus | 47 | 14% |  |
| Number of open-ended items with novel responses | 25 | 8% |  |
| *Omissions on the survey* |  |  |  |
| Number of open ended responses used because a minimum frequency was not provided | 36 | 11% |  |
| Number of open ended responses used because a "Don't know" option was not provided | 21 | 6% |  |
| Number of open ended responses that were available in the dropdown menu | 44 | 13% |  |
| Number who wrote "I brought it from home" | 47 | 14% |  |
| Number who wrote "I don't want to" | 157 | 47% |  |
