# Supplementary material for: Electronic based reported anthropometry—A useful tool for interim monitoring of obesity prevalence in developing states
Source: PLoS One. 2020 Dec 7;15(12):e0243202. doi: 10.1371/journal.pone.0243202 (PMC7721176; doi:10.1371/journal.pone.0243202)
Supplement: S3 File — (DOCX) [file pone.0243202.s004.docx]

**The Barbados Children’s Health and Nutrition Study**

**The St. Michael School Canteen Pilot**

**R-Code that was used for the Support Vector Machine (SVM)**

**#packages install**

install.packages("tidyverse",dependencies = T)

install.packages("dplyr",dependencies = T)

install.packages("caret",dependencies = T)

library(tidyverse)

library(dplyr)

library(caret)

**#Data slicing for caret**

set.seed(3333)

intrain <- createDataPartition(y = working_dataset3$weight_status_code, p= 0.7, list = FALSE)

training <- working_data_file[intrain,]

testing <- working_data_file[-intrain,]

**#Check the dimension of the training and testing set**

dim(training); dim(testing);

**#Preprocessing & Training**

anyNA(working_data_file)

**#Dataset summarized details**

training[["dependent_variable"]] = factor(training[["dependent_variable "]])

**#Training the SVM model**

trctrl <- trainControl(method = "repeatedcv", number = 10, repeats = 5)

set.seed(3333)

**#SVM Classifier using Non-Linear Kernel**

svm_Radial_kernal <- train(dependent_variable ~., data = training, method = "svmRadial",

trControl=trctrl,

preProcess = c("center", "scale"),

tuneLength = 10)

**#To see the results of the SVM,**

svm_Radial_kernal

**#Plot showing that our classifier is giving best accuracy**

plot(svm_Radial_kernal)

test_pred_Radial <- predict(svm_Radial_kernal, newdata = testing)

testing$weight_status_code<-as.factor(testing$weight_status_code)

confusionMatrix(test_pred_Radial, testing$weight_status_code )

**# Tuning the Sigma**

grid_radial <- expand.grid(sigma = seq(0.1,100,0.2),

C = 2^(2:7))> set.seed(3233)

svm_Radial_Grid

plot(svm_Radial_Grid)

test_pred_Radial_Grid <- predict(svm_Radial_Grid, newdata = testing)

confusionMatrix(test_pred_Radial_Grid, testing$weight_status_code)
