# Supplementary material for: Electronic based reported anthropometry—A useful tool for interim monitoring of obesity prevalence in developing states
Source: PLoS One. 2020 Dec 7;15(12):e0243202. doi: 10.1371/journal.pone.0243202 (PMC7721176; doi:10.1371/journal.pone.0243202)
Supplement: S4 File — (DOCX) [file pone.0243202.s005.docx]

**St. Michael’s School Canteen Pilot Study**

**Focus Group Guide**

**PART 1 - Introduction to the Discussion**

Reintroduce yourself to the participants and thank them for agreeing to participate in the focus group.

Ask the students to sit comfortably in a semicircle so that all are able to see each other’s faces during the discussion.

**The Reason for Conducting the Focus Group**

Tell the participants the following:

I’m going to ask you some questions about the canteen survey that you recently completed

The information you give me will be used to help improve the way in which we deliver questionnaires to you

Do remember that you are free to speak and make any comments you wish to. These will be kept confidential.

You gave me your permission to take detailed notes of your answers to these questions. This will help us to accurately report what you wish to say. We will write the answers out but your name will never be used. No one at your school will have access to the notes.

**Explanation of How the Session Will be Conducted**

Let me explain to you how I’d like us to conduct this discussion

1. Everything you say is important
2. Everyone will be given a chance to speak
3. Remember that no one else will hear what you say so you can speak freely
4. I would be grateful if you would turn off your cell phones

**The Discussion**

*Barriers to recruitment*

1.     Did you hear anything about the canteen survey going on in your school?

2.     Was this at assembly or otherwise?

3.     Did you listen to the Researcher from UWI?

4.     Did the announcements from the principal and senior researcher at full assembly influence your decision to engage in the survey?

a.     Did this encourage you to complete the survey?

*Reasons for not completing the survey*

5.     Did you receive an e-mail from you school e-mail address requesting that you complete a canteen survey?

a. Did you open the internet page?

b. Did you attempt the survey?

c. I remembered my log on credentials

d. I check my school e-mail/I use e-mail very often

6.     I understood the questions on the survey?

8.     Did your parents refuse permission?

9.  Did you understand how to use the drop-down menu?

10.  I stopped the survey before the end because it was too long

11.  Many of you responded using the free-answer option. Why?

a.     Did you often want to say things that were not available in the drop-down lists

12.  Did you have any concerns?

*Recommendations for improvement*

13.  Have you any suggestions as to what would encourage you to answer the survey?

**PART 3 - Ending The Focus Group Session**

We have come to the end of the formal question and answer section but you may give us any additional comments you wish to.

I wish to thank you all for coming.
