# Supplementary material for: Electronic based reported anthropometry—A useful tool for interim monitoring of obesity prevalence in developing states
Source: PLoS One. 2020 Dec 7;15(12):e0243202. doi: 10.1371/journal.pone.0243202 (PMC7721176; doi:10.1371/journal.pone.0243202)
Supplement: S5 File — (DOCX) [file pone.0243202.s006.docx]

**PLEASE RETURN WITH YOUR SIGNATURE**

**Consent Statement:**

I understand that participation in this study is completely voluntary.

⁪ Yes, I allow my child ________________________________________________ to take part in this study.

(child’s name)

⁪ No, I do not allow my child to take part in this study.

Signature of Parent or Guardian:________________________________

Date: ______________________________

(dd/mm/yyyy)

**Investigators:** Pamela Gaskin, PhD

Nutrition Epidemiologist

Principal Investigator

**Co-investigator:** Peter Chami, PhD

Statistician

You are invited to participate in the The St. Michael’s School Canteen Study. The main objective of this study is to describe the relationship of canteen food consumption to desirability as assessed by adolescents

and changes in food consumptions patterns among adolescents with changes in food offerings.

Participation in this study will require your child to have weight and height measured. In addition, he/she will complete an electronic survey on foods offered by the canteen at their school.

This study is being conducted by the University of the West Indies and is endorsed by the The Ministry of Education, Technological and Vocational Training. Your child was selected as a possible participant because he/she is in the age range we are interested in studying. We ask that you read this form carefully. You are welcome to ask any questions you may have before agreeing to have your child in this study.

**Neither you nor your child will be placed in harmful or compromising situations.**

The identity of your child will be kept confidential and secure. Only the principal and data manager will have access to the identifying information. The data collected will remain stored for at least 3 years on an electronic database and shall be used for research or policy purposes.

Participation in this study is voluntary. One may withdraw if he/she wishes as well as refuse to answer any questions. Your decision whether or not to participate or to allow for your child’s participation will not affect current or future relations with his/her school. While there are no direct benefits to you or your child, we hope that findings from this study will help with policymaking.

**_____________________________________**

Pamela Gaskin PhD

Principal Investigator

Lecturer, Essential National Health Research

If you have any questions or concerns about your child's rights or welfare as a participant in this research study, or for more information please contact the following:

| **Principal Investigator**  Pamela Gaskin, Ph.D.  Faculty of Medical Sciences,  University of the West Indies, Cave Hill  Email: [pamela.gaskin@cavehill.uwi.edu](mailto:pamela.gaskin@cavehill.uwi.edu)  Telephone: **(246) 820 0105** / (246) 271 1510 |  |
| --- | --- |
| Peter Chami, Ph.D.  Faculty of Science and Technology  University of the West Indies, Cave Hill  Email: [peter.chami@protonmail.com](mailto:peter.chami@protonmail.com) |  |
|  |  |
|  |  |

Any further concerns may be addressed to The Institutional Review Board of the University of the West-Indies Cave Hill/Ministry of Health Barbados at (246) 417 4847.
