# Supplementary material for: Electronic based reported anthropometry—A useful tool for interim monitoring of obesity prevalence in developing states
Source: PLoS One. 2020 Dec 7;15(12):e0243202. doi: 10.1371/journal.pone.0243202 (PMC7721176; doi:10.1371/journal.pone.0243202)
Supplement: Attachment — Submitted filename: Response to Reviewers.doc [file pone.0243202.s012.doc]

**IN REPLY PLEASE QUOTE**

**OUR REFERENCE:**

September 23, 2020,

Academic Editor
PLOS ONE

Dear Dr. Lombardo,

We thank the reviewers for their cogent comments to our submission “Electronic-based reported anthropometry - A useful tool for interim monitoring of obesity prevalence in developing states*”.* We have attempted to revise and improve the manuscript using this guidance. Edits are highlighted using Track Changes in Microsoft Word directly in the text. Below we have responded to the comments of the editor and the reviewers in the order in which they were made. Your comments are in Italics and our responses in normal type.

**ACADEMIC EDITOR COMMENTS TO AUTHOR**

1. *“Thank you for submitting your manuscript to PLOS ONE. After careful consideration, we feel that it has merit but does not fully meet PLOS ONE’s publication criteria as it currently stands. Therefore, we invite you to submit a revised version of the manuscript that addresses the points raised during the review process.”*

We have made every attempt to comply with the publication criteria of PLOS ONE. As such, we have responded to reviewers and added additional supporting material.

**REVIEWER COMMENTS:**

1. *If the authors have adequately addressed your comments raised in a previous round of review and you feel that this manuscript is now acceptable for publication, you may indicate that here to bypass the “Comments to the Author” section, enter your conflict of interest statement in the “Confidential to Editor” section, and submit your "Accept" recommendation.*

*Reviewer #1: All comments have been addressed*

*Reviewer #2: All comments have been addressed*

2. *Is the manuscript technically sound, and do the data support the conclusions?

   The manuscript must describe a technically sound piece of scientific research with data that supports the conclusions. Experiments must have been conducted rigorously, with appropriate controls, replication, and sample sizes. The conclusions must be drawn appropriately based on the data presented.

   Reviewer #1: Yes
   Reviewer #2: Partly*

We have added the raw data from the electronic survey in the supporting information in a file labelled “The Electronic Survey raw data”.

*3. Has the statistical analysis been performed appropriately and rigorously?

Reviewer #1: I Don't Know
Reviewer #2: No*

We have reviewed the statistical analysis and we believe it to be appropriate and rigorous.

4. *Have the authors made all data underlying the findings in their manuscript fully available?

The [PLOS Data policy](http://www.plosone.org/static/policies.action#sharing) requires authors to make all data underlying the findings described in their manuscript fully available without restriction, with rare exception (please refer to the Data Availability Statement in the manuscript PDF file). The data should be provided as part of the manuscript or its supporting information or deposited to a public repository. For example, in addition to summary statistics, the data points behind means, medians and variance measures should be available. If there are restrictions on publicly sharing data—e.g. participant privacy or use of data from a third party—those must be specified.

Reviewer #1: (No response)
Reviewer #2: No*

We have added additional supporting information giving the raw data for the electronic survey and the age/sex distribution of all students in the school in files labelled “The Electronic Survey raw data” and “List of all children by form sex and dob” respectively.

5. Is the manuscript presented in an intelligible fashion and written in standard English?

PLOS ONE does not copyedit accepted manuscripts, so the language in submitted articles must be clear, correct, and unambiguous. Any typographical or grammatical errors should be corrected at revision, so please note any specific errors here.

Reviewer #1: Yes

Reviewer #2: (No Response)

6. Review Comments to the Author

*Please use the space provided to explain your answers to the questions above. You may also include additional comments for the author, including concerns about dual publication, research ethics, or publication ethics. (Please upload your review as an attachment if it exceeds 20,000 characters)*

*Reviewer #1: This revised manuscript provided answers to important issues. Major comments have been addressed. So, I hope to accept this manuscript.*

*Reviewer #2: The reviewer appreciates the responses and has a couple questions.

First, the* ***survey is still not included.*** *The survey should be included.

Second, the response rate was* ***very low****. What the authors have actually shown is that the sample that responded had reasonable agreement with the measurements. What are the characteristics of the sample that did not respond? If the authors cannot demonstrate that the individuals who did not respond are different at least in base characteristics, they cannot make a strong statement that all adolescents can supply height and weight by survey.*

1. We apologize for this omission. We have added the raw data from the electronic survey in the file “The Electronic Survey raw data” in the supporting information.
2. While we admit that the response rate to the survey was low, we have no reason to assume that those who did not answer were unable to supply weight and height by electronic survey. We had baseline age and sex for each student and we have included results at **line 293** that which demonstrate that the students who answered the electronic survey were from the same distribution as those in the entire school. At **line 425** we discussed this. In addition, do recall that on the reliability study, where we had a representative sample of the school, all children were able to give a credible estimate of their weight and height. We have no reason to believe that they would not have been able to write down these same reported weights and heights on an electronic survey. Also, during the focus groups none of the students reported difficulty with understanding how to answer the survey as a reason for not responding to the electronic survey.

*7. PLOS authors have the option to publish the peer review history of their article ([what does this mean?](https://journals.plos.org/plosone/s/editorial-and-peer-review-process#loc-peer-review-history)). If published, this will include your full peer review and any attached files.


If you choose “no”, your identity will remain anonymous but your review may still be made public.*


***Do you want your identity to be public for this peer review?*** *For information about this choice, including consent withdrawal, please see our* [*Privacy Policy*](https://www.plos.org/privacy-policy)*.*

*Reviewer #1: No*

*Reviewer #2: Yes:* *Diana Thomas*

*[NOTE: If reviewer comments were submitted as an attachment file, they will be attached to this email and accessible via the submission site. Please log into your account, locate the manuscript record, and check for the action link "View Attachments". If this link does not appear, there are no attachment files.]

While revising your submission, please upload your figure files to the Preflight Analysis and Conversion Engine (PACE) digital diagnostic tool,* [*https://pacev2.apexcovantage.com/*](https://pacev2.apexcovantage.com/)*. PACE helps ensure that figures meet PLOS requirements. To use PACE, you must first register as a user. Registration is free. Then, login and navigate to the UPLOAD tab, where you will find detailed instructions on how to use the tool. If you encounter any issues or have any questions when using PACE, please email PLOS at* [*figures@plos.org*](mailto:figures@plos.org)*. Please note that Supporting Information files do not need this step.*

*In compliance with data protection regulations, you may request that we remove your personal registration details at any time.* [*(Remove my information/details)*](https://www.editorialmanager.com/pone/login.asp?a=r)*. Please contact the publication office if you have any questions.*

Yours faithfully,

Pamela Gaskin PhD

Lecturer, Essential National Health Research
