# Supplementary material for: Electronic based reported anthropometry—A useful tool for interim monitoring of obesity prevalence in developing states
Source: PLoS One. 2020 Dec 7;15(12):e0243202. doi: 10.1371/journal.pone.0243202 (PMC7721176; doi:10.1371/journal.pone.0243202)
Supplement: Attachment — Submitted filename: Response to Reviewers.doc [file pone.0243202.s013.doc]

**IN REPLY PLEASE QUOTE**

**OUR REFERENCE:**

October 12, 2020,

Academic Editor
PLOS ONE

Dear Dr. Lombardo,

We thank the reviewers for their cogent comments to our submission “Electronic-based reported anthropometry - A useful tool for interim monitoring of obesity prevalence in developing states*”.* We have attempted to revise and improve the manuscript using this guidance. Edits are highlighted using Track Changes in Microsoft Word directly in the text. Below we have responded to the comments of the editor and the reviewers in the order in which they were made. Your comments are in Italics and our responses in normal type.

Reviewers' comments:

Reviewer's Responses to Questions

**Comments to the Author**

*1. If the authors have adequately addressed your comments raised in a previous round of review and you feel that this manuscript is now acceptable for publication, you may indicate that here to bypass the “Comments to the Author” section, enter your conflict of interest statement in the “Confidential to Editor” section, and submit your "Accept" recommendation.*

*Reviewer #2: All comments have been addressed*

*2. Is the manuscript technically sound, and do the data support the conclusions?

The manuscript must describe a technically sound piece of scientific research with data that supports the conclusions. Experiments must have been conducted rigorously, with appropriate controls, replication, and sample sizes. The conclusions must be drawn appropriately based on the data presented.*

*Reviewer #2: Yes*

*3. Has the statistical analysis been performed appropriately and rigorously?*

*Reviewer #2: Yes*

*4. Have the authors made all data underlying the findings in their manuscript fully available?

The [PLOS Data policy](http://www.plosone.org/static/policies.action#sharing) requires authors to make all data underlying the findings described in their manuscript fully available without restriction, with rare exception (please refer to the Data Availability Statement in the manuscript PDF file). The data should be provided as part of the manuscript or its supporting information, or deposited to a public repository. For example, in addition to summary statistics, the data points behind means, medians and variance measures should be available. If there are restrictions on publicly sharing data—e.g. participant privacy or use of data from a third party—those must be specified.*

*Reviewer #2: Yes*

*5. Is the manuscript presented in an intelligible fashion and written in standard English?

PLOS ONE does not copyedit accepted manuscripts, so the language in submitted articles must be clear, correct, and unambiguous. Any typographical or grammatical errors should be corrected at revision, so please note any specific errors here.*

*Reviewer #2: (No Response)*

*6. Review Comments to the Author

Please use the space provided to explain your answers to the questions above. You may also include additional comments for the author, including concerns about dual publication, research ethics, or publication ethics. (Please upload your review as an attachment if it exceeds 20,000 characters)*

*Reviewer #2: It's not satisfactory to say that we have no reason to believe there is no difference between those who filled the survey or not. Make a table of the non-survey takers and the survey takers with statistical characteristics. Is BMI different between groups? Even better is to run a t-test.*

We addressed the comparison of BMI differences among those who filled the survey compared to non-survey takers at **line 296** in the results section. Given that we did not have weights and heights for the entire school population we used BMIs reported on the reliability study, which had a representative sample of the school, to compare with the BMIs reported in the electronic survey. It is reasonable to expect that the distribution of the BMI on the representative sample would be very similar to that of the school.

Of the 71 students who answered the electronic survey, 65 reported weights and heights, compared to 270 students on the reliability study. We carried out an independent samples t-test to assess whether the mean reported BMI on the electronic survey was significantly different from that of the representative sample. There was no significant difference in BMI between the two groups (Mean difference; 1.49; *p* = 0.203).

Do recall that a priori we expected a low response rate so that the electronic survey was never intended to be used as a means of assessing children’s ability to report anthropometry reliably. Rather it was used to explore ability to complete the survey and to help us to understand barriers and solutions to completion of surveys presented in this format.

7. PLOS authors have the option to publish the peer review history of their article ([what does this mean?](https://journals.plos.org/plosone/s/editorial-and-peer-review-process#loc-peer-review-history)). If published, this will include your full peer review and any attached files.


If you choose “no”, your identity will remain anonymous but your review may still be made public.


**Do you want your identity to be public for this peer review?** For information about this choice, including consent withdrawal, please see our [Privacy Policy](https://www.plos.org/privacy-policy).

Reviewer #2: No

[NOTE: If reviewer comments were submitted as an attachment file, they will be attached to this email and accessible via the submission site. Please log into your account, locate the manuscript record, and check for the action link "View Attachments". If this link does not appear, there are no attachment files.]

While revising your submission, please upload your figure files to the Preflight Analysis and Conversion Engine (PACE) digital diagnostic tool, <https://pacev2.apexcovantage.com/>. PACE helps ensure that figures meet PLOS requirements. To use PACE, you must first register as a user. Registration is free. Then, login and navigate to the UPLOAD tab, where you will find detailed instructions on how to use the tool. If you encounter any issues or have any questions when using PACE, please email PLOS at [figures@plos.org](mailto:figures@plos.org). Please note that Supporting Information files do not need this step.

Yours sincerely

Pamela Gaskin PhD

Lecturer, Essential National Health Research
